# Dihydroartemisinin Induces Ferroptosis in Uveal Melanoma Cells Through the HO-1 and xCT/GPX4 Signaling Pathways

**DOI:** 10.3390/ijms27073027

**Published:** 2026-03-26

**Authors:** Yuxuan Zhao, Chen Hou, Lirong Xiao, Longqian Liu, Naihong Yan

**Affiliations:** 1 Research Laboratory of Ophthalmology, West China Hospital, Sichuan University, Chengdu 610041, China; zhaoyx413@stu.wchscu.cn (Y.Z.); xiaolirong@wchscu.cn (L.X.); 2 Department of Ophthalmology, West China Hospital, Sichuan University, Chengdu 610041, China; houchen323@wchscu.cn (C.H.); liulq@scu.edu.cn (L.L.); 3 Research Laboratory of Macular Disease, West China Hospital, Sichuan University, Chengdu 610041, China

**Keywords:** uveal melanoma, natural product, ferroptosis, heme oxygenase 1

## Abstract

Uveal melanoma (UM) represents an uncommon intraocular malignancy with high aggressiveness. Dysregulation of ferroptosis has been associated with UM progression. Dihydroartemisinin (DHA), a natural derivative of *Artemisia annua*, exhibits potent antitumor activity with a favorable safety profile, yet its role in ferroptosis regulation in UM remains unclear. Here, we showed that DHA significantly reduced the proliferation and invasiveness of UM cells—both primary and secondary—with effects intensifying over time and dose. Transcriptomic analysis indicated that DHA may exert antitumor effects by modulating the ferroptosis-related pathway, characterized by upregulating heme oxygenase-1 (HO-1) and downregulating the SLC7A11 (xCT)/GPX4 axis, leading to iron accumulation, increased ROS and lipid peroxidation, and mitochondrial dysfunction. Iron chelators and pancaspase inhibitors partially reverse these effects, whereas HO-1 inducers enhance them. Overall, our results suggest that DHA suppresses UM progression by inducing ferroptosis and mitochondrial dysfunction, while the HO-1 and xCT/GPX4 pathways may contribute to these effects. DHA may represent a potential therapeutic approach for UM, warranting further investigation.

## 1. Introduction

As a rare but extremely lethal primary intraocular tumor, uveal melanoma (UM) has a strong tendency to metastasize [1]. Its incidence varies by geographic region and ethnicity [2]. Current treatment options for primary UM include local excision, radiation therapy (such as plaque radiation therapy and proton beam therapy), and enucleation. Although these treatments have shown clinical efficacy, they often result in visual impairment or even blindness, significantly affecting patients’ quality of life [3]. Moreover, more than half of UM patients eventually develop distant metastasis. A meta-analysis revealed that the median survival time after metastasis is only 10–13 months, with an extremely poor prognosis and no curative treatment available [4]. These clinical challenges highlight the urgency of searching for innovative therapeutic options that can effectively target both primary and metastatic UM with minimal adverse effects.

As a novel form of regulated cell death, ferroptosis is characterized by its reliance on iron metabolism and oxidative lipid damage. It initiates when an imbalance occurs among the ferroptosis-driving forces and the cellular protective mechanisms [5]. Under physiological conditions, cells preserve intracellular redox homeostasis by eliminating lipid peroxides. However, when lipid peroxides accumulate excessively or when the cellular defense system is impaired, ferroptosis is triggered [6]. Recent research findings indicate a strong correlation between cellular ferroptosis defense gene expressions and the prognosis and risk prediction in patients with UM [7], suggesting that targeting ferroptosis represents a promising therapeutic approach.

Recently, a growing number of natural products are emerging as valuable sources of anticancer agents [8]. Artemisinin (ART), a compound derived from the plant *Artemisia annua* L., is primarily recognized for its antimalarial properties [9]. Dihydroartemisinin (DHA), the semisynthetic compound of ART, exhibits notable antitumor efficacy across multiple cancers, including lymphocytic leukemia, cervical cancer, and liver cancer [10]. Owing to their favorable safety profile and potent antitumor activity, ART and its derivatives are considered as potential therapies to cancer treatment. At present, several clinical trials are being conducted to assess ART and its derivatives as adjuvant therapies for drug-resistant tumors [11,12].

ART has also been identified as a potent ferroptosis inducer with therapeutic relevance in clinical oncology [13]. These compounds trigger ferroptosis by elevating free iron concentrations within cells and promoting lipid peroxide accumulation, thereby enhancing the efficacy of anticancer treatments [14,15,16]. Prior research indicates that ART compounds elevate intracellular iron concentrations by promoting ferritin degradation through the lysosomal pathway, thereby inducing ferroptosis in cancer cells [17]. Additionally, ART and its derivatives disrupt mitochondrial respiratory function by affecting the electron transport chain, which leads to increased production of reactive oxygen species (ROS) [18].

The mechanisms by which DHA induces ferroptosis in UM remain unexplored. Here, we examined the phenotypic impact of DHA on UM cells and identified the molecular mechanism underlying DHA-induced ferroptosis, thereby offering mechanistic insights into its therapeutic potential.

## 2. Results

### 2.1. DHA Suppresses the Proliferation, Migration, and Invasion of UM Cells

**Figure 1:**
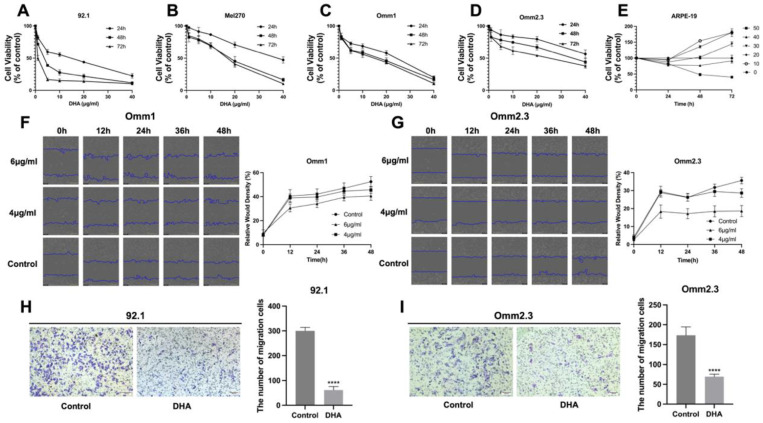
Dihydroartemisinin (DHA) inhibits uveal melanoma (UM) cell proliferation, migration, and invasion. (**A**–**D**) The viability of primary (92.1, Mel270) and metastatic (Omm1, Omm2.3) UM cell lines were assessed using a CCK-8 assay following treatment with varying concentrations of DHA (0–40 μg/mL) for 24, 48, and 72 h. (**E**) The results of the CCK-8 assay revealed that DHA (0–40 μg/mL) had no notable cytotoxic effect on ARPE-19 cells. (**F**,**G**) Cell migration was assessed using a wound healing assay in Omm1 and Omm2.3 cells following treatment with 4 or 6 μg/mL DHA for 12, 24, 36, or 48 h. Scale bar = 400 μm (**H**,**I**) Transwell invasion assays were conducted to assess the invasive capacity of 92.1 (3 μg/mL) and Omm2.3 (40 μg/mL) cells after 48 h of DHA treatment. Scale bar = 100 μm. **** indicates statistical significance, *p* < 0.0001.

The CCK-8 assay revealed that DHA significantly decreased the viability of both primary and metastatic UM cells, the effect being dose- and time-dependent-sensitive. The suppression of cell viability became more evident after 48 h of DHA treatment. At 48 h, DHA exhibited varying cytotoxicity across UM cell lines, with IC_50_ (half maximal inhibitory concentration, IC_50_) values of 3.12 for 92.1, 15.47 for Mel270, 11.61 for Omm1, and 40.09 for Omm2.3 (μg/mL, mean ± SD, *n* = 5). Among them, 92.1 cells were the most sensitive, whereas Omm2.3 cells showed the greatest resistance. Notably, in ARPE-19 cells, low concentrations of DHA (0–40 μg/mL) did not affect cell viability, indicating a favorable safety profile for normal retinal cells (Figure 1E).

Live-cell imaging over a 0–48 h period demonstrated that DHA significantly restricted UM cell migration, with higher concentrations producing more pronounced inhibitory effects (*p* < 0.001 for 4 μg/mL; Figure 1F,G). Transwell invasion assays further demonstrated that DHA significantly decreased the quantity of invading UM cells (*p* < 0.001, *n* = 3; Figure 1H,I). Overall, DHA inhibited UM cell growth, migration, and invasion in a dose- and time-dependent manner, while exerting minimal cytotoxicity on normal retinal cells.

### 2.2. DHA Promotes UM Cell Death by Regulating the HO-1 and xCT/GPX4 Signaling Pathways

**Figure 2:**
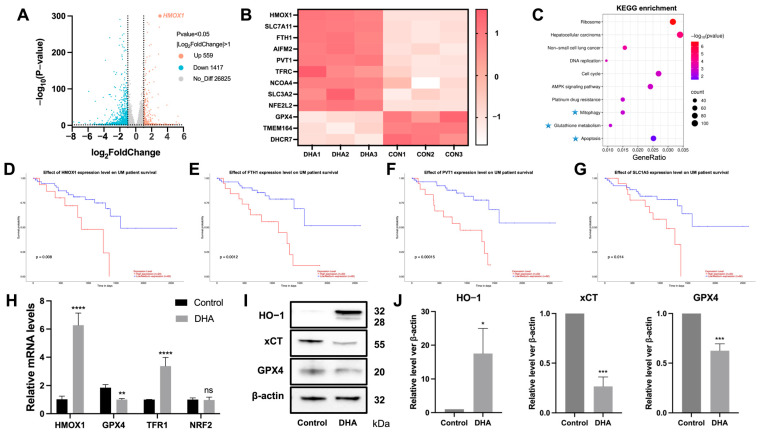
DHA induces UM cell death by modulating the heme oxygenase-1 (HO-1) and SLC7A11 (xCT)/GPX4 signaling pathways. (**A**) Volcano plot showing genes whose expression differed between Omm1 cells treated with DHA (15 μg/mL) and control cells, with thresholds of |log_2_FC| > 1 and *p* < 0.05. (**B**) Heatmap illustrating the expression profiles of selected ferroptosis-related genes in the DHA-treated samples versus the control samples. (**C**) KEGG pathway enrichment analysis revealing significant involvement of glutathione metabolism, mitochondrial, and apoptosis-related pathways. ✭ indicates pathways that may be involved in the action of DHA. (**D**–**G**) Kaplan-Meier survival curves from the TCGA datasets demonstrating that high expression of ferroptosis-related genes (*HMOX1*, *FTH1*, *PVT1*, and *SLC1A5*) is correlated with poor survival in UM patients. (**H**) qRT-PCR analysis of the gene expression of *HMOX1*, *GPX4*, *TFR1*, and *NRF2*. (**I**,**J**) Western blot analysis of HO-1, xCT, and GPX4 protein expression levels. The protein concentration was normalized to that of β-actin. **** indicates statistical significance, *p* < 0.0001. *** indicates statistical significance, *p* < 0.001. ** indicates statistical significance, *p* < 0.01. * indicates statistical significance, *p* < 0.05, ns means not significant.

To investigate the molecular mechanisms underlying DHA-induced UM cell death, transcriptomic sequencing and bioinformatics analyses were performed. We identified 1976 differently expressed genes (DEGs), of which 559 were upregulated and 1417 were downregulated (|log_2_FC| ≥ 1, adjusted *p* < 0.05; Figure 2A). Genes associated with ferroptosis were significantly enriched; among these genes, the expression of *HMOX1* and *FTH1* was upregulated, and that of the oxidative stress-related gene *GPX4* was downregulated (Figure 2B). Furthermore, we performed KEGG pathway enrichment analysis to investigate the biological processes underlying the DEGs identified in DHA-treated UM cells. KEGG pathway analysis revealed enrichment in ferroptosis and related pathways, including the glutathione (GSH) mitochondrial pathways (*p* adjusted < 0.05; Figure 2C). As a central component of the ferroptosis metabolic pathway, GSH modulates ferroptosis by regulating GPX4 activity. Moreover, ferroptosis is closely associated with mitochondrial damage induced by ROS. Notably, pathways involved in various cancers were enriched, including hepatocellular carcinoma, non-small cell lung cancer, and glioma. These pathways could offer insights into the broader context of DHA’s effect in cancer cell death. Additionally, TCGA data analysis revealed notable associations between ferroptosis-associated genes (including *HMOX1*, *FTH1*, *PVT1*, *SLC1A5*) and patients’ survival outcomes (*p* < 0.05; Figure 2D–G). The findings suggest that ferroptosis might be a key process underlying DHA-induced UM cell death.

On the basis of these observations, several ferroptosis-associated genes were selected for validation. qRT-PCR demonstrated a significant increase in *HMOX-1* expression (6.3 ± 0.3-fold vs. control, *p* < 0.001), a decrease in antioxidant enzyme *GPX4* expression (0.5 ± 0.3-fold, *p* < 0.01), and an increase in transferrin receptor *TFR1* expression (3.3 ± 0.3-fold, *p* < 0.001), whereas *NRF2* expression remained unchanged (*p* > 0.05; Figure 2H). Western blot analysis validated these transcriptional alterations at the protein level, demonstrating consistent increases in HO-1 expression alongside the suppression of SLC7A11 (xCT) and GPX4 (mean ± SD, *n* = 3, *p* < 0.05; Figure 2I,J). Collectively, these results indicate that DHA-induced UM cell death is accompanied by upregulation of HO-1 and suppression of the xCT/GPX4 axis, suggesting that these pathways may be involved in this process.

### 2.3. DHA Induces Ferroptosis in UM Cells

**Figure 3:**
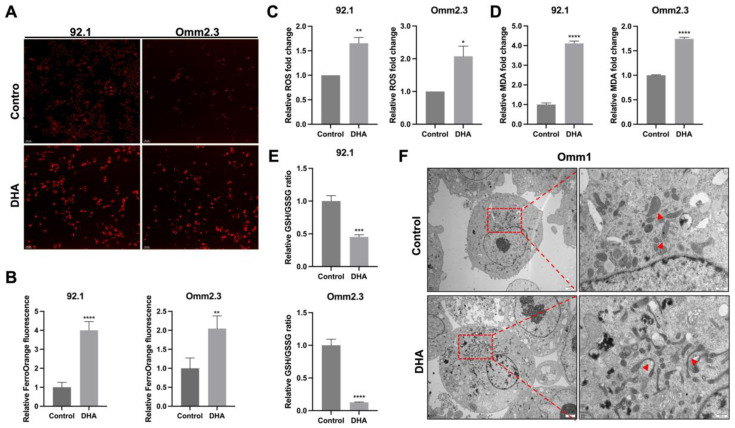
DHA induces ferroptosis in UM cells. (**A**,**B**) Intracellular ferrous ion (Fe^2+^) levels were evaluated by FerroOrange staining in 92.1 (3 μg/mL) and Omm2.3 (40 μg/mL) cells following 48 h of DHA treatment. Scale bar = 70 μm (**C**) Intracellular reactive oxygen species (ROS) levels were measured using a DCFH-DA probe in 92.1 and Omm2.3 cells after 48 h of treatment. (**D**) Malondialdehyde (MDA) levels, an indicator of lipid peroxidation, were quantified using an MDA assay kit. (**E**) The intracellular glutathione (GSH)/GSSG ratio was determined using a GSSG/GSH Quantification Kit II. (**F**) Transmission electron microscopy revealed characteristic ferroptotic features, including condensed mitochondria, loss of cristae, and increased membrane density (highlighted by red arrowheads), in the DHA-treated (15 μg/mL) Omm1 cells. Scale bar = 20 μm for the two images on the left, and Scale bar = 500 nm for the two images on the right. **** indicates statistical significance, *p* < 0.0001. *** indicates statistical significance, *p* < 0.001. ** indicates statistical significance, *p* < 0.01. * indicates statistical significance, *p* < 0.05.

Iron catalyzes ferroptosis, characterized by lipid peroxidation and compromised cell membranes. To experimentally validate the role of ferroptosis during the DHA-induced suppression on UM cells, multiple ferroptosis-related indicators were assessed. Intracellular ferrous ion (Fe^2+^) levels were visualized using FerroOrange, which revealed an elevated Fe^2+^ level in the DHA-treated cells (*p* < 0.05; Figure 3A,B), likely due to HO-1-mediated heme degradation. Excessive Fe^2+^ within cells catalyzed the Fenton reaction, generating elevated levels of ROS, as confirmed by increased DCF fluorescence intensity using a DCFH-DA probe (*p* < 0.01; Figure 3C).

Additionally, GSH and malondialdehyde (MDA) levels, representing antioxidant capacity and lipid peroxidation respectively, were assessed with commercial kits. After 48 h of DHA treatment, the GSH levels significantly decreased (*p* < 0.05, *n* = 3), whereas the MDA levels markedly increased (*p* < 0.001, *n* = 3), indicating lipid peroxidation and oxidative stress (Figure 3D,E). Transmission electron microscopy revealed classical morphological features of ferroptosis in the DHA-treated cells, including condensed mitochondria, loss of cristae, and increased membrane density (Figure 3F). Collectively, the findings demonstrate DHA-triggered ferroptosis in UM cells.

### 2.4. Mitochondrial Damage Is Associated with DHA-Induced Ferroptosis in UM Cells

**Figure 4:**
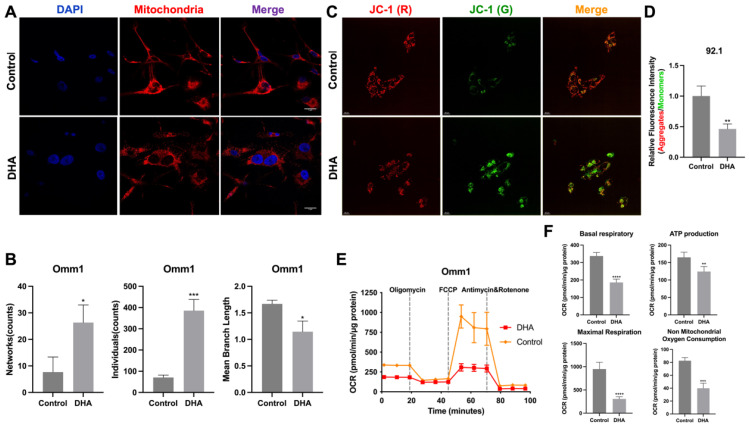
DHA-induced ferroptosis is associated with mitochondrial damage in UM cells. (**A**) Mitochondrial morphology was visualized using a Mito Red fluorescent probe in Omm1 cells after 48 h of DHA (15 μg/mL) treatment. Scale bar = 5 μm. (**B**) Quantification of mitochondrial parameters, including the number of individual mitochondria, network structures, and mean branch length were analyzed using the macro-MINA tool in ImageJ. (**C**) MMP was evaluated using JC-1 staining in 92.1 cells after 24 h of DHA (3 μg/mL) treatment. Scale bar = 20 μm. (**D**) The ratio of red (aggregated) to green (monomeric) JC-1 fluorescence intensity was calculated to assess the MMP integrity. (**E**) The mitochondrial oxygen consumption rate (OCR) was measured using a Seahorse XF Analyzer following sequential injection of oligomycin, FCCP, and antimycin A/rotenone in Omm1 cells after treatment with DHA (15 μg/mL). (**F**) Quantified respiratory parameters included basal respiration, ATP production, maximal respiration, and nonmitochondrial oxygen consumption in control versus DHA-treated cells. **** indicates statistical significance, *p* < 0.0001. *** indicates statistical significance, *p* < 0.001. ** indicates statistical significance, *p* < 0.01. * indicates statistical significance, *p* < 0.05.

Mitochondria, central to metabolic processes, are also involved in ferroptosis and apoptosis regulation through the mitochondrial permeability transition pores [19,20]. To further elucidate mitochondrial structural and functional changes during DHA-induced ferroptosis, mitochondrial staining, a JC-1 assay, and a mitochondrial respiratory stress test were performed. Mito Red staining indicated an elevated mitochondria quantity within individuals and networks, accompanied by a reduction in the average branch length (*p* < 0.05, Figure 4A,B). Mitochondrial dysfunction, often indicated by Mitochondrial membrane potential (MMP) loss, was assessed using JC-1 staining. Mitochondria with intact MMP show red JC-1 aggregates, while loss of MMP causes JC-1 to persist as green-fluorescent monomers. A red-to-green fluorescence shift observed via JC-1 staining reflects the loss of the MMP, a hallmark of early apoptosis (*p* < 0.01, *n* = 3; Figure 4C,D).

Mitochondrial respiratory function was further assessed using a Seahorse XF Analyzer. DHA significantly suppressed the oxygen consumption rate (OCR) (*p* < 0.01, *n* = 3), indicating impaired oxidative phosphorylation activity (Figure 4E,F). Collectively, the evidence supports the finding that DHA promotes ferroptosis in UM cells through pronounced alterations in mitochondrial morphology and energy metabolism.

### 2.5. Pharmacological Inhibition of Ferroptosis and Apoptosis Partially Attenuates DHA-Induced UM Cell Death

**Figure 5:**
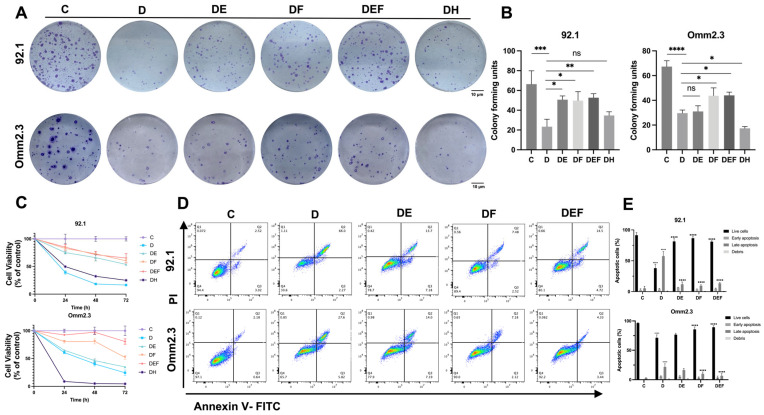
Pharmacological inhibition of ferroptosis and apoptosis partially attenuates DHA-induced UM cell death. (**A**,**B**) Colony formation assays were conducted in 92.1 (3 μg/mL DHA) and Omm2.3 (40 μg/mL DHA) cells treated with DHA alone or in combination with various pharmacological inhibitors for 48 h. Compared with treatment with DHA alone, cotreatment with the ferroptosis inhibitor DFO (10 μg/mL) or the apoptosis inhibitor EMR (10 μg/mL) partially restored colony formation, whereas cotreatment with hemin (10 μg/mL) further reduced the clonogenic potential. Scale bar = 10 μm. (**C**) Cell viability was assessed at 24, 48, and 72 h. Combined treatment with DFO or EMR moderately improved survival, whereas cotreatment with hemin further accelerated cell death. (**D**,**E**) Apoptotic cell populations were assessed by flow cytometry after 48 h of treatment. The proportions of live cells, early apoptosis, late apoptosis, and debris were reduced in the groups treated with ferroptosis or apoptosis inhibitors in combination with DHA. Colors indicate cell density, with blue representing low density, green representing medium density, and red representing high density. Different quadrants represent different types of apoptotic cells: Q1, debris; Q2, late apoptotic cells; Q3, early apoptotic cells; and Q4, live cells. (**** *p* < 0.0001 vs. C, ■■■■ *p* < 0.0001 vs. D). **** indicates statistical significance, *p* < 0.0001. *** indicates statistical significance, *p* < 0.001. ** indicates statistical significance, *p* < 0.01. * indicates statistical significance, *p* < 0.05. ns means not significant.

To investigate potential involvement of alternative regulated cell death pathways, ferroptosis, apoptosis, necroptosis, and autophagy inhibitors were used. Ferrostatin-1 (Fer-1), necrostatin-1, and 3-methyladenine showed no protective effect against DHA-induced cell death (Supplementary Figure S1A). In contrast, deferoxamine (DFO) and emricasan (EMR) improved cell survival to some extent, implicating ferroptosis and apoptosis in DHA-triggered cytotoxicity (Figure 5A–C). The experimental groups were defined as follows: C, control; D, DHA alone; DE, DHA + EMR; DF, DHA + DFO; DEF, DHA + EMR + DFO; and DH, DHA + Hemin.

Hemin, an oxidized form of hemoglobin and a known inducer of HO-1 expression, was further used to explore the role of HO-1. CCK-8 and colony formation experiments revealed that coadministration of DHA and hemin further enhanced UM cell death, indicating that HO-1 overexpression exacerbated DHA-induced cytotoxicity (*p* < 0.05, *n* = 3; Figure 5A–C).

Mechanistic validation using FerroOrange staining and flow cytometry further supported these findings. Live-cell imaging revealed that DFO chelated intracellular Fe^2+^ and thereby reduced iron-mediated oxidative damage, ultimately attenuating DHA-induced cell death (*p* < 0.05, *n* = 3; Supplementary Figure S1B). Flow cytometric analysis indicated that EMR and DFO partially decreased the proportion of late apoptotic cells (*p* < 0.01, *n* = 3; Figure 5D,E). Notably, this protective effect was more prominent in the 92.1 cells when EMR was used alone. Together, these findings suggest that both ferroptosis and apoptosis synergistically mediate DHA-induced cell death in UM, and the interplay between these two mechanisms may contribute to the overall effect.

## 3. Discussion

As the leading primary intraocular tumor in adults, UM is characterized by high rates of metastasis and recurrence, often posing a significant threat to patients’ health and quality of life. Current treatment strategies are limited, and no effective targeted therapies have been approved to date [21]. Therefore, identifying novel therapeutic agents is an urgent clinical priority. DHA, a semisynthetic derivative of ART, exhibits broad-spectrum antitumor activity with low toxicity [13]. Prior studies show that ART suppresses UM progression by regulating Wnt/β-catenin and PI3K/AKT/mTOR pathways [22,23]. The current research further clarified the antitumor role of DHA in UM by inducing ferroptosis.

In this study, DHA significantly inhibited the proliferation of both primary as well as metastatic UM cell lines but exhibited minimal toxicity to normal retinal pigment epithelial cells. Functional assays, including wound healing and Transwell invasion assays, further confirmed that DHA effectively suppressed UM cell migration and invasion ability.

Research evidence indicates that xCT and GPX4 expression, key regulators of ferroptosis, is significantly linked to prognosis and risk assessment in UM [7]. In a zebrafish model of UM, the induction of ferroptosis has been shown to reduce the incidence of metastatic disease [7]. Thus, targeting ferroptosis emerges as a potential treatment option for UM based on these observations.

Our transcriptomic analysis suggested that DHA promotes ferroptosis in UM cells, likely via HMOX1 activation and regulation of the xCT/GPX4 antioxidative system. The TCGA dataset demonstrated that ferroptosis-related gene expression is prognostically relevant in UM, supporting a potential ferroptosis-driven mechanism. On the basis of these observations, our study focused primarily on elucidating the mechanisms by which DHA triggers ferroptosis.

**Figure 6:**
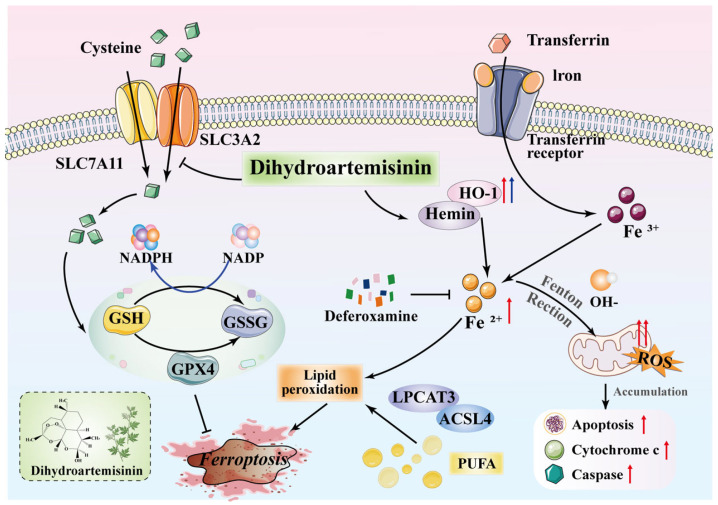
Molecular mechanisms through which DHA targets ferroptosis in UM cells. DHA is extracted from the leaves of *Artemisia annua* Linn. The xCT/SLC3A2-GSH-GPX4 system plays a critical role in cellular resistance to ferroptosis. In UM cells, DHA promotes ferroptosis by inhibiting the xCT/GPX4 signaling pathway. In the catabolic products of heme and HO-1, divalent iron ions are generated through the Fenton reaction, which accelerates the accumulation of lipid peroxides and ROS, further promoting ferroptosis. Additionally, the Fenton reaction produces highly reactive hydroxyl radicals (OH-), which, under the catalytic effects of OH- and enzymes such as ACSL4 and LPCA3, initiate the lipid peroxidation chain reaction. PUFAs trigger the lipid peroxidation chain reaction. This cascade ultimately leads to ferroptosis. DHA facilitates ferroptosis primarily through the upregulation of HO-1 and downregulation of the xCT/GPX4 signaling pathway, two key steps that promote ferroptosis. The iron chelator deferoxamine (DFO) prevents iron-dependent lipid peroxidation by sequestering free iron. The red and blue arrows next to HO-1 indicate its dual role: elevated HO-1 can both promote cell survival and induce cell death.

Ferroptosis, a newly recognized form of regulated cell death, involves ROS excess, iron deposition, and lipid peroxidation [24]. Among these features, intracellular iron buildup and lipid peroxidation are regarded as primary triggers of ferroptosis [25]. Under DHA treatment, HO-1 overexpression accelerates heme degradation, leading to substantial Fe^2+^ release. When the intracellular iron transport system fails to eliminate this excess iron, the Fenton reaction generates high levels of ROS. ROS oxidize membrane polyunsaturated fatty acids (PUFAs), producing abundant lipid peroxides. The resulting oxidative damage disrupts membrane integrity and ultimately triggers ferroptosis (Figure 6).

xCT functions as a solute carrier in amino acid translocation via the plasma membrane. Specifically, xCT facilitates cystine uptake and glutamate release, supporting intracellular GSH synthesis. This system combats oxidative damage, maintains redox balance, and prevents lipid peroxidation-induced cell death [26]. In this study RNA-seq analysis showed increased mRNA levels of *SLC7A11* and *SLC3A2* after DHA treatment, whereas Western blot analysis demonstrated decreased protein expression of these transporters. This discrepancy may reflect post-transcriptional regulation, altered translational efficiency, or reduced protein stability under DHA treatment. Indeed, changes at the mRNA level do not always correlate with protein abundance. The increased transcription of *SLC7A11* and *SLC3A2* may represent a compensatory cellular response to oxidative stress, while DHA may simultaneously suppress the accumulation of the corresponding proteins by affecting their translation or promoting their degradation. GPX4 is an intracellular selenium-containing antioxidant enzyme that detoxifies membrane-associated lipid hydroperoxides. By utilizing GSH, GPX4 reduces phospholipid hydroperoxides derived from polyunsaturated fatty acids into nontoxic phospholipid alcohols (PLOHs), thereby suppressing lipid peroxidation and preventing ferroptosis [27]. The xCT-GSH-GPX4 axis constitutes the core of the cellular defense system against ferroptosis [28,29,30]. This study revealed that DHA downregulates this axis, leading to GSH depletion and GPX4 inactivation, resulting in increased MDA accumulation and impaired redox homeostasis. The collapse of this defense system renders UM cells highly susceptible to ferroptosis (Figure 6).

The iron chelator DFO and pancaspase inhibitor EMR partially reversed DHA-induced UM cell death. Intracellular iron accumulation occurs through transferrin receptor-mediated uptake. Intracellular iron overload drives the Fenton reaction, producing ROS that induce lipid peroxidation, damage DNA and proteins, and ultimately trigger ferroptosis [31]. DFO chelates free iron, thereby blocking the Fenton reaction and directly inhibiting key ferroptotic processes. Although unique, ferroptosis may engage in crosstalk with apoptosis depending on certain microenvironment. For instance, ROS buildup in ferroptosis can elevate MMP, trigger cytochrome c release, and activate caspase-dependent apoptosis [32]. In this context, EMR might mitigate DHA-induced cytotoxicity by inhibiting caspase-dependent pathways.

Our findings demonstrate that DHA induces cell death through both ferroptosis and apoptosis. While initial experiments suggested that ferroptosis might be the dominant mechanism, as evidenced by the significant inhibition of cell death by DFO, contradictory results were observed when using Fer-1, which did not show protective effects. Several possible explanations may account for this discrepancy:

(1) Iron dependency of ferroptosis: As an iron-dependent process, ferroptosis induced by DHA is largely mediated through iron overload and Fenton reaction. DFO directly chelates excess Fe^2+^, thereby blocking the source of ROS upstream. In contrast, Fer-1 acts only on downstream lipid peroxidation and cannot resolve iron overload; thus, it fails to fully inhibit ferroptosis.
(2) Cell type-specific pharmacodynamics: The ineffectiveness of Fer-1 in UM cells may result from limited stability or poor cellular permeability. Fer-1 may be rapidly degraded or fail to enter UM cells effectively [33]. In contrast, DFO acts extracellularly and intracellularly to chelate iron, and its efficacy is not constrained by membrane permeability or stability issues.
(3) Stage-specific ferroptosis: DHA-induced ferroptosis in UM cells involves advanced or irreversible mitochondrial damage. If ferroptosis has progressed to a late stage involving mitochondrial disintegration, Fer-1, which acts downstream, may not be able to repair structural damage. In contrast, by intervening upstream, DFO may prevent further oxidative injury and mitochondrial collapse.

These findings emphasize the importance of selecting stage-appropriate and mechanism-targeted inhibitors when intervening in ferroptosis-associated cell death.

Our apoptosis-related assays suggest that apoptosis may not be the only form of cell death induced by DHA. The relatively lower proportion of Q2 cells (late apoptosis) in Omm2.3 suggests that apoptosis alone may not fully account for DHA-induced cell death. Combined with the observed alterations in lipid peroxidation and ferroptosis-related molecules, these findings raise the possibility that other non-apoptotic cell death mechanisms also contribute to the cytotoxic effects of DHA. And this dual mechanism raises important questions regarding the interplay between ferroptosis and apoptosis in DHA-treated cells. While our data indicate that both pathways are involved, the precise contribution of each remains unclear. Further studies, including genetic knockdown experiments, will be necessary to dissect the relative roles of ferroptosis and apoptosis in this context. Importantly, these findings underscore the complexity of cell death mechanisms triggered by DHA and highlight the need for a more nuanced understanding of how ferroptosis and apoptosis interact to drive cellular outcomes.

HO-1 is a critical antioxidant enzyme that degrades heme into biliverdin, CO, and Fe^2+^. Although generally regarded as cytoprotective, HO-1 has also been implicated in promoting tumor progression across various cancers. It reduces chemotherapy-induced oxidative stress, suppresses apoptosis and autophagy, and enhances tumor cell growth and metastasis [34]. Importantly, in our previous study, we demonstrated that HO-1 plays a crucial role in uveal melanoma progression, as both pharmacological inhibition with ZnPP and genetic silencing of HO-1 markedly suppressed UM cell growth and induced apoptosis [35]. Consistent with this concept, studies in other tumor types have shown that ferroptosis can be induced through downregulating the Nrf2/HO-1/GPX4 axis [36]. In addition, accumulating evidence suggests that HO-1 may exert a dual role in ferroptosis. Under certain conditions, excessive HO-1 activation may promote ferroptotic cell death by increasing intracellular Fe^2+^ levels and oxidative stress beyond the buffering capacity of ferritin [37]. In the present study, DHA treatment upregulated HO-1 expression in UM cells, and pharmacological activation of HO-1 with hemin further enhanced DHA-induced cell death, suggesting that the HO-1 axis may be involved in DHA-triggered ferroptosis [38]. While moderate HO-1 expression provides cytoprotection, whereas excessive upregulation can surpass ferritin buffering, causing Fe^2+^ overload and promoting ferroptotic death [39]. However, this conclusion should be interpreted with caution. Unlike our previous work, the present study did not include HO-1-specific genetic knockdown or overexpression experiments to directly verify whether HO-1 is functionally required for DHA-induced ferroptosis. Therefore, our current data primarily support the involvement of the HO-1 pathway, rather than definitively establishing HO-1 dependency in this process. Future studies incorporating genetic approaches will be necessary to further clarify the precise role of HO-1 in DHA-induced ferroptosis in UM cells.

In the current therapeutic landscape of uveal melanoma, tebentafusp and darovasertib represent the most significant clinical advances. Tebentafusp, a bispecific TCR fusion protein, has been approved as the first systemic therapy for metastatic UM and demonstrated a median overall survival of approximately 21.7 months in phase III clinical trials [40]. However, its clinical benefits are largely restricted to HLA-A*02:01-positive patients, and resistance often develops over time. Darovasertib, a selective protein kinase C (PKC) inhibitor, has shown encouraging activity in early-phase trials, with disease control rate of around 90% when used alone or in combination with c-Met inhibitors such as crizotinib [41]. Nevertheless, its efficacy remains limited, and acquired resistance is a frequent challenge.

ART, licensed in China for malaria treatment, has undergone randomized clinical evaluation in breast and colorectal cancer, confirming its safety and tolerability [11,42]. ART was used as a therapeutic option in two patients with metastatic UM for whom conventional chemotherapy failed, resulting in extended median survival [43]. Furthermore, clinical studies show that prolonged high-dose ART treatment exhibits minimal systemic toxicity and favorable safety [12]. Importantly, ferroptosis inducers such as DHA and other ferroptosis inducers offer mechanistically distinct alternatives by promoting iron-dependent lipid peroxidation and disrupting redox homeostasis [44]. This unique mechanism could complement existing therapies and potentially overcome resistance associated with PKC inhibition or immune-based approaches [21]. However, this translational potential should be interpreted with caution. Ferroptosis inducers have been associated with cardiovascular and neurological risks, raising safety concerns that must be carefully addressed before clinical application [21]. Beyond efficacy, several key challenges remain to be resolved for the clinical translation of DHA. First, the systemic toxicity profile and pharmacokinetic characteristics of DHA in UM remain insufficiently defined, particularly with regard to long-term exposure and off-target effects. Second, the poor water solubility and rapid metabolic clearance of ART derivatives pose significant delivery challenges, highlighting the need for optimized drug formulations or nanoparticle-based delivery systems to improve bioavailability and tumor targeting. Third, although partial in vivo validation has demonstrated the therapeutic potential of ART derivatives, the available in vivo data remain limited in scope. Rigorous preclinical studies are required to delineate the therapeutic window, establish optimal dosing strategies, and unsure sustained safety.

Additionally, there are two remaining limitations in the methodology of this study. First, the use of low-risk cell lines like Omm1 in our transcriptional study may limit the generalizability of the findings to metastatic stages of uveal melanoma. Future studies incorporating metastatic cell lines such as Omm2.3 will be essential to better understand the full therapeutic potential of DHA and its efficacy across different UM stages. In addition, the present study did not include genetic or inhibitory experiments targeting SLC7A11/xCT or GPX4. Therefore, although our results suggest that the xCT/GPX4 axis is involved in DHA-induced ferroptosis, the current evidence remains preliminary and does not definitively establish pathway dependency.

## 4. Materials and Methods

### 4.1. Cell Culture

Human UM cell lines (92.1, Mel270, Omm1, Omm2.3) and ARPE-19 cells were obtained from reported sources [25,45]. Specifically, the human uveal melanoma cell line Omm1 was generously provided by Professor M. J. Jager of Leiden University Medical Centre and Professor Renbing Jia from the Department of Ophthalmology, Ninth People’s Hospital, Shanghai Jiao Tong University. Omm2.3 and Mel270 cell lines were obtained from the Institute of Ophthalmology, West China Hospital, Sichuan University. The 92.1 cell line was purchased from Wuhan Pricella Biotechnology Co., Ltd (Wuhan, China). Human retinal pigment epithelial cells, ARPE-19, were kindly provided by Dr. Kang Zhang from West China Hospital, Sichuan University. Dihydroartemisinin (DHA) was purchased from MedChemExpress (MCE, Cat. No. HY-N0176, CAS No. 71939-50-9). Product details are available on the MedChemExpress website: https://www.medchemexpress.cn/Dihydroartemisinin.html (accessed on 23 March 2026).

Cells were maintained in RPMI 1640 (Thermo Scientific, Waltham, MA, USA) or DMEM/F12 (Thermo Scientific, Waltham, MA, USA) with 10% FBS (ExCell Bio, Chengdu, China) and 1% penicillin–streptomycin (Thermo Scientific, Waltham, MA, USA) at 37 °C in 5% CO_2_.

### 4.2. Cell Viability Assay

Cell viability was assessed by CCK-8 assay (MCE, Monmouth Junction, NJ, USA). Cells were seeded in 96-well plates, exposed to DHA or DMSO, and incubated for 24–72 h. After adding CCK-8 reagent for 1 h, absorbance at 450 nm was measured. IC_50_ values were calculated with GraphPad Prism 10.

### 4.3. Wound Healing Assay

Cell migration was evaluated by a wound healing assay. Cells were seeded in 96-well plates, scratches were introduced with an Incucyte WoundMaker, and images acquired over 0–48 h using the Incucyte S3 system (Sartorius, Göttingen, Germany). Relative wound density (%) was calculated as the ratio of cell density within the wound area at each time point to that in the area outside the wound in the same field of view, using the built-in analysis software (version 07, 2022).

### 4.4. Transwell Invasion Assay

Cell invasion was assessed in Transwell chambers (8 μm, Jet Biofil, Guangzhou, China). Cells were seeded in serum-free medium, with 20% FBS in the lower chamber. After 48 h, migrated cells were fixed, crystal violet-stained, and quantified by ImageJ (version 1.54g).

### 4.5. RNA Sequencing

RNA sequencing was performed by Novogene (Beijing, China) to identify differentially expressed genes (DEGs). Total RNA was extracted using TRIzol, and its quality was assessed prior to sequencing. RNA-seq libraries were prepared and sequenced on the Illumina NovaSeq 6000 platform (Illumina, San Diego, CA, USA), with a minimum sequencing depth of 30 million paired-end reads per sample.

The reads were aligned to the reference genome using HISAT2 (version 2.2.1), and gene expression counts were generated using feature Counts. Data normalization was performed using TPM (transcripts per million), and differential expression was analyzed with DESeq2 (version 1.36.0), applying the Benjamini–Hochberg FDR method for multiple-testing correction. Genes with *p*-value below 0.05 and an absolute log_2_fold change of at least 1 were further analyzed by KEGG enrichment.

### 4.6. Real-Time Quantitative Polymerase Chain Reaction

qRT-PCR was performed to quantify mRNA expression. Total RNA was extracted (Foregene, Chengdu, China) and analyzed in triplicate, with GAPDH as the reference control. Detailed primer sequences can be found in Supplementary Table S1.

### 4.7. Western Blot

Protein expression was assessed by Western blot. Cell lysates were prepared in RIPA buffer, quantified by BCA, and equal protein (25 μg) was separated by SDS-PAGE and transferred to PVDF membranes. Blots were incubated with antibodies against HO-1 (AB189491, Abcam, Cambridge, UK), xCT (A25302, ABclonal, Wuhan, China), GPX4 (A27995, ABclonal, Wuhan, China), and β-actin (EM21002, Huabio, Hangzhou, China), followed by HRP-conjugated secondary antibodies (HA1001 and HA1006, Huabio, Hangzhou, China). Bands were visualized with ECL and analyzed using ImageJ.

### 4.8. Transmission Electron Microscopy

Transmission electron microscopy (TEM) was performed to assess cellular ultrastructure. Cells were fixed with 3% glutaraldehyde and processed at the Lilai Medical Research Centre (Chengdu, China) for embedding, sectioning, and imaging.

### 4.9. Fe^2+^ Level Measurement

Intracellular Fe^2+^ was measured using FerroOrange (Dojindo, Kumamoto Prefecture, Kyushu Island, Japan) in DHA-treated cells, rinsed with HBSS, and incubated with 1 μM FerroOrange. Fluorescence images were captured by microscope and quantified with ImageJ.

### 4.10. Detection of Intracellular ROS Levels

Intracellular ROS was measured with the DCFH-DA probe (Beyotime, Shanghai, China) post-DHA treatment, followed by probe incorporation. Fluorescence was detected with a microplate reader (Ex 488 nm, Em 525 nm).

### 4.11. Measurement of GSH and MDA Levels

GSH and MDA were measured using a GSSG/GSH Quantification Kit II and an MDA Assay Kit (Dojindo, Kumamoto Prefecture, Kyushu Island, Japan). After reaching optimal confluence, cells were exposed to DHA treatment, and metabolite levels were determined following the manufacturers’ guidelines.

### 4.12. Mito Red Staining and Mitochondrial Morphology Analysis

Mitochondrial morphology was assessed with Mito Red staining (Dojindo, Kumamoto Prefecture, Kyushu Island, Japan) in DHA-treated cells, followed by fixation, DAPI counterstaining, and antifade mounting. Fluorescence images were acquired microscopically. The analysis of mitochondrial number, network, and mean branch length was conducted using the macro-MINA tool (version 1.8.1), developed for mitochondrial network analysis within ImageJ. The tool can be accessed at: github.com/ScienceToolkit/MiNA. The analysis procedure is as follows: in ImageJ, select an individual mitochondrial structure, then navigate to Plugins > Macros > MiNA > Analyze Mitochondrial Morphology to begin the analysis.

### 4.13. Mitochondrial Membrane Potential

MMP was assessed with JC-1 kit (Beyotime, Shanghai, China). Cells were plated, exposed to DHA, stained with 1 mM JC-1, and analyzed by fluorescence microscopy and ImageJ.

### 4.14. Mitochondrial Respiration Mito Stress Test

Mitochondrial respiration was evaluated using the Seahorse XFe24 Mitochondrial Stress Test (Agilent Technologies, Santa Clara, CA, USA) according to the protocol described in [46].

### 4.15. Colony Formation Assay

Colony formation assays assessed low-density proliferation. Cells were treated with DHA, DFO, EMR, or hemin, and after 14–21 days, colonies were fixed, stained, and quantified with ImageJ.

### 4.16. Apoptosis

Apoptosis was assessed with an Annexin V-FITC/PI kit (Simu Biotechnology, Shanghai, China) after treating cells with DHA, DFO, or EMR. Stained cells were analyzed by flow cytometry using FlowJo (version 10.8.1).

### 4.17. Statistical Analysis

Statistical analyses were performed using GraphPad Prism 10, with results presented as mean ± SD. T tests or one-way ANOVA followed by Tukey’s multiple comparison test were applied. Each experiment was repeated at least three times. Significance was set at *p* < 0.05 (* *p* < 0.05, ** *p* < 0.01, *** *p* < 0.001, **** *p* < 0.0001).

## 5. Conclusions

In conclusion, this study suggests that DHA induces ferroptosis and apoptosis in UM cells, accompanied by disrupted iron metabolism and impaired antioxidant defenses. Our data indicate that DHA treatment is associated with increased HO-1 expression, iron accumulation, and ROS elevation, as well as suppression of the xCT/GPX4 pathway, which may collectively contribute to enhanced lipid peroxidation and ferroptotic vulnerability in UM cells. Although these findings support DHA as a potentially effective ferroptosis-inducing agent in UM, the precise functional roles of the HO-1 and xCT/GPX4 pathways require further validation. Future comparative studies against current standard-of-care therapies, such as tebentafusp and darovasertib, together with comprehensive preclinical evaluations of systemic toxicity, pharmacokinetics, and delivery strategies, will be essential to further assess the translational potential of DHA for UM treatment.

## Data Availability

Prognostic evaluation of ferroptosis-associated genes in UM was conducted using UALCAN (http://ualcan.path.uab.edu/) (accessed on 23 March 2026), an open-access platform enabling comprehensive TCGA-based analyses. The complete RNA-seq dataset are available upon request from the corresponding author via email (yannaihong@wchscu.cn).

## Supplementary Materials

The following supporting information can be downloaded at: https://www.mdpi.com/article/10.3390/ijms27073027/s1.

## Abbreviations

The following abbreviations are used in this manuscript:

UM	Uveal melanoma
DHA	Dihydroartemisinin
HO-1	heme oxygenase-1
ART	Artemisinin
ROS	reactive oxygen species
IC_50_	half maximal inhibitory concentration
DEGs	differently expressed genes
GSH	glutathione
MDA	malondialdehyde
MMP	Mitochondrial membrane potential
OCR	oxygen consumption rate
Fer-1	Ferrostatin-1
DFO	Deferoxamine
EMR	Emricasan
PKC	protein kinase C
TEM	Transmission electron microscopy
