# Supplementary figures and images for: Dihydroartemisinin Induces Ferroptosis in Uveal Melanoma Cells Through the HO-1 and xCT/GPX4 Signaling Pathways
Source: Int J Mol Sci. 2026 Mar 26;27(7):3027. doi: 10.3390/ijms27073027 (PMC13073528; doi:10.3390/ijms27073027)

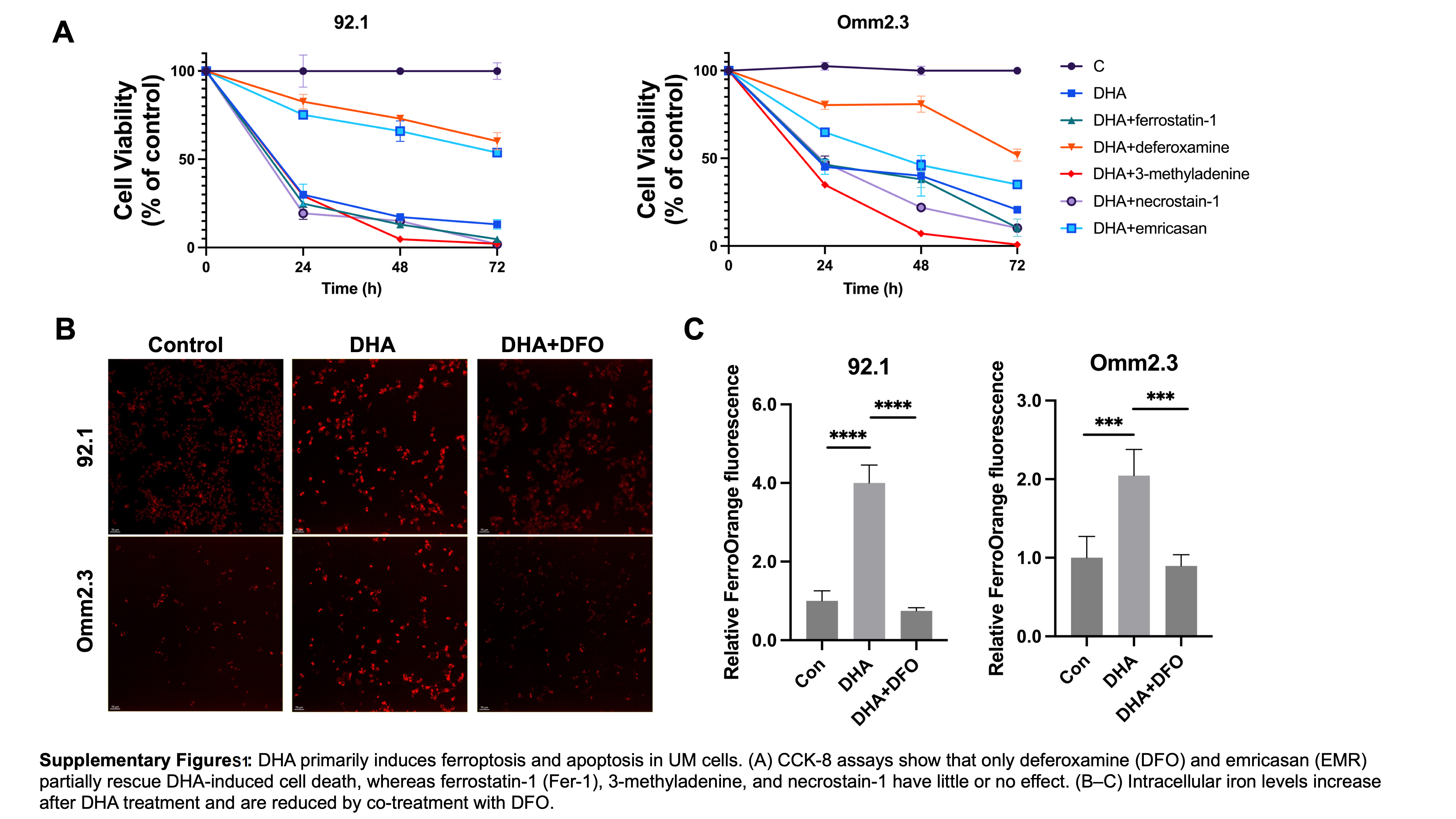

Supplement: Supplementary file 1 [file ijms-27-03027-s001.zip › Supplementary Figure S1.tiff]
